# Impact of ocean acidification on the hypoxia tolerance of the woolly sculpin, *Clinocottus analis*

**DOI:** 10.1093/conphys/cow040

**Published:** 2016-10-04

**Authors:** Joshua R. Hancock, Sean P. Place

**Affiliations:** Sonoma State University, Department of Biology, Rohnert Park, CA 94928,USA

**Keywords:** Hypoxia, metabolic rate, ocean acidification, sculpin, stress response

## Abstract

As we move into the Anthropocene, organisms inhabiting marine environments will continue to face growing challenges associated with changes in ocean pH (ocean acidification), dissolved oxygen (dead zones) and temperature. These factors, in combination with naturally variable environments such as the rocky intertidal zone, may create extreme physiological challenges for organisms that are already performing near their biological limits. Although numerous studies have examined the impacts of climate-related stressors on intertidal animals, little is known about the underlying physiological mechanisms driving adaptation to ocean acidification and how this may alter organism interactions, particularly in marine vertebrates. Therefore, we have investigated the effects of decreased ocean pH on the hypoxia response of an intertidal sculpin, *Clinocottus analis*. We used both whole-animal and biochemistry-based analyses to examine how the energetic demands associated with acclimation to low-pH environments may impact the fish's reliance on facultative air breathing in low-oxygen environments. Our study demonstrated that acclimation to ocean acidification resulted in elevated routine metabolic rates and acid–base regulatory capacity (Na^+^,K^+^-ATPase activity). These, in turn, had downstream effects that resulted in decreased hypoxia tolerance (i.e. elevated critical oxygen tension). Furthermore, we present evidence that these fish may be living near their physiological capacity when challenged by ocean acidification. This serves as a reminder that the susceptibility of teleost fish to changes in ocean pH may be underestimated, particularly when considering the multiple stressors that many experience in their natural environments.

## Abstract (precis)

Global climate change may exacerbate naturally occurring stressors encountered by intertidal organisms. This in turn, may reduce the ability of these organisms to withstand the daily stress of life in the intertidal zone. Here we investigated how elevated CO_2_ might impact low oxygen tolerance of a marine teleost.

## Introduction

Anthropogenically driven global climate change is fundamentally altering ocean environments at an alarming rate (Doney *et al*., 2009). As atmospheric carbon dioxide concentrations continue to rise above 400 μatm, the invariably linked consequences of a warmer planet and more acidic oceans are becoming more evident. In the past 50 years alone, average global temperatures have increased by ~0.6°C (Walther *et al*., 2002), while oceanic carbon dioxide sequestration has occurred at a rate 100 times greater than at any time in the past 650 000 years (Siegenthaler *et al*., 2005). The intergovernmental panel on climate change (IPCC) predicts that by the year 2100, our oceans could decrease in pH by 0.3–0.4 units, corresponding to a partial pressure of carbon dioxide ($P_{{\mathrm{CO}}_{2}}$) upwards of 1000 μatm (IPCC, 2013). Indeed, as we monitor these broad-scale environmental changes, it becomes apparent that marine organisms are, and will continue to be, faced with physiological stressors associated with both increased sea surface temperatures (ocean warming) and declining ocean pH (ocean acidification; Hughes, 2000; Fabry *et al*., 2008).

Regional differences in environmental conditions will be likely to affect the impact of climate-related stressors on marine biota. For example, ocean acidification may exacerbate high-$P_{{\mathrm{CO}}_{2}}$ conditions associated with upwelling in coastal oceans (Hales *et al*., 2005; Hauri *et al*., 2009; Thomsen *et al*., 2010; Gruber, 2011). Likewise, ocean acidification will probably act in combination with other environmental variables (e.g. temperature, salinity) that are naturally variable in the rocky intertidal zone to create extreme physiological challenges for organisms that may already be performing near their biological limits. Numerous studies have demonstrated that within the intertidal zone, small changes in environmental conditions could have consequences for fitness and community composition, while also causing distributional shifts as the adaptive capacities of these organisms are pushed to their limits (Helmuth *et al*., 2006; Tomanek, 2008). Subsequently, it becomes important to investigate the physiological and biochemical pathways challenged by the exacerbating effects of climate change in intertidal organisms in order to understand whole-organism and ecosystem consequences.

Unlike temperature stress, relatively little is known about the underlying physiological mechanisms driving adaptation to ocean acidification, particularly in vertebrates (Kroeker *et al*., 2010). Perhaps one reason for this is that teleost fishes have been thought to be resilient to intermediate levels of increasing carbon dioxide because of their well-established acid–base regulatory capacity (e.g. Ishimatsu *et al*., 2005, 2008). However, the maintenance of cellular homeostasis comes at a significant energetic cost (Kültz, 2005; Hochachka and Somero, 2014). As nearly all cellular functions rely on strict homeostasis of intracellular pH, fishes must expend large amounts of energy regulating ion concentrations. The largest portion of pH maintenance is done via chloride cells in the gill epithelium, which rely on Na^+^,K^+^-ATPase (NKA) pumps in concert with carbonic anhydrase, a ubiquitous enzyme found in vertebrates, to catalyse the hydrolysis reaction of CO_2_ and enhance the exchange of Na^+^ and Cl^−^ for H^+^ and HCO_3_^−^ (Clairborne *et al*., 2002; Gilmour and Perry, 2009). At higher carbon dioxide concentrations, NKA pumps can be up-regulated in response to excess H^+^ ions in the cell (Ishimatsu *et al*., 2005; Deigweiher *et al*., 2008). The high costs of these basal functions are thought to translate into differences in species distribution and behaviour and, ultimately, underlie trade-offs in fitness, especially in intertidal organisms (Somero, 2002; Lang *et al*., 2009). Indeed, shifts in energy allocation have previously been observed in teleost fish in response to hypercapnic conditions where acid–base balance can consume upwards of 40% of the total cellular energy budget (Deigweiher, 2009). Although the study by Deigweiher (2009) used severe hypercapnia (~10 000 ppm CO_2_), it is likely that ecologically relevant changes in $P_{{\mathrm{CO}}_{2}}$, combined with additional stresses such as hypoxia or ocean warming, will result in energy shifts and create fitness trade-offs in teleost fishes.

Intertidal sculpins in the genus *Clinocottus*, such as the woolly sculpin (*Clinocottus analis*), are common to the coast of central California. These fishes, like most intertidal organisms, experience a variety of abiotic stressors brought about by low tide, including depleted oxygen content (hypoxia) in tide pools with a high degree of respiration (Yoshiyama, 1981). When oxygen becomes limiting, sculpins may use behavioural mechanisms, such as facultative aerial respiration, to satisfy metabolic needs (Martin, 1991; Watters and Cech, 2003). With an increased demand for oxygen to maintain cellular function in more acidic waters, intertidal sculpins capable of aerial respiration may exit hiding spots in their tide pools for longer periods of time. This behaviour, however, has a trade-off, as sculpins face increased temperature, desiccation and predation near the surface and outside of the water. Conversely, in response to deoxygenation, oxygen-expensive cellular machinery such as NKA pumps may be down-regulated (Bogdanova *et al*., 2005) in order to conserve oxygen and avoid exiting their tide pool. By doing so, however, sculpins may fail to defend against the accumulation of H^+^ and become vulnerable to acidosis. This trade-off leads to an interesting juxtaposition when these behavioural and physiological responses to hypoxia are considered in conjunction with the projected decreases in ocean pH associated with ocean acidification. The acclimatory response of these fish to future $P_{{\mathrm{CO}}_{2}}$ levels should involve a significant up-regulation of the acid–base regulatory pathway and incur an elevated energetic cost. This long-term acclimatory response, however, would also be expected to impair the sculpin's ability to compensate physiologically for low-oxygen environments via down-regulation of energetically expensive pathways as described above. Instead, long-term declines in pH may require the sculpin to rely on behavioural alterations such as facultative respiration to offset the energy demands, which could increase predation risk for sculpins in these ecosystems.

Here, we investigated the potential for ocean acidification to alter the routine metabolic rate (RMR), critical oxygen tension ($P_{O_{2}\mathrm{crit}}$, a marker for hypoxia tolerance), and acid–base regulatory capacity in the gill of *C. analis*. In addition, we attempted to gain insight into potential behavioural changes in these fish by monitoring the oxygen tension at which fish first displayed facultative aerial respiration. To date, only a handful of studies have examined the effects of ocean acidification on teleost physiology, and none, that we are aware of, have been conducted on fishes living within the intertidal zone. Furthermore, even fewer have quantified acid–base regulatory capacity or were able to draw insight from changes to whole-animal physiology, which are relevant to ecologically important behaviours, such as hypoxia avoidance. Our understanding of how global climate change may impact the rocky intertidal community is limited by an incomplete understanding of pH effects on intertidal fishes, which act as important members of the intertidal food web. We hypothesize that the decreases in ocean pH associated with ocean acidification would challenge these animals physiologically as evidenced by increases in basal metabolism, decreases in hypoxia tolerance and increased activity of NKA pumps as an indicator of acid–base regulatory capacity.

## Materials and methods

### Collection of study specimens

Woolly sculpins, *C. analis*, commonly found in tide pools along the California coast, were collected from Horseshoe Cove (38.316, −123.071) and Shell Beach (38.417, −123.107) near Bodega Bay, CA, USA. Specimens from the mid-intertidal pools were caught during low tide on three different dates, 28 July (*n* = 12), 1 October (*n* = 12) and 23 November 2015 (*n* = 14), using dip nets. All fish were transported in fresh aerated seawater to recirculating seawater aquaria at Sonoma State University, where they were maintained in ambient seawater conditions (~14°C, 400 μatm $P_{{\mathrm{CO}}_{2}}$) for at least 1 week prior to the start of experimentation. Fish were fed to satiation every other day using a diet of frozen mysis and brine shrimp. All fish were handled according to guidelines approved by the Sonoma State University Institutional Animal Care and Use Committee (protocol # 2015-51).

### Experimental design

After acclimation to the aquaria for at least 7 days, fish were randomly distributed to one of 16 10-litre experimental tanks that consisted of a control treatment that mimicked the ambient seawater conditions near Horseshoe Cove (~400 μatm $P_{{\mathrm{CO}}_{2}}$, 14°C) or an elevated CO_2_ treatment (~1100 μatm $P_{{\mathrm{CO}}_{2}}$, 14°C) that was consistent with predicted future ocean conditions (IPCC, 2013). Fish were placed in experimental tanks for a period of 7 days (*n* = 12 per treatment) or 28 days (*n* = 7 per treatment) to examine the effects of ocean acidification on the RMR, $P_{O_{2}\mathrm{crit}}$ and Na^+^,K^+^-ATPase enzyme activity. It was noticed prior to experimentation that *C. analis* individuals could be territorial. Thus, to prevent behavioural altercations, no more than two fish were housed per aquarium, and all fish were separated by a physical barrier that prevented contact between fish but allowed water flow throughout the aquarium.

### Manipulation of seawater conditions

Treatment $P_{{\mathrm{CO}}_{2}}$ levels were maintained using a $P_{{\mathrm{CO}}_{2}}$-generating system, described by Fangue *et al*. (2010) and further modified for the use on fish by Enzor *et al*. (2013). Briefly, atmospheric air was dried using a drying column of drierite and scrubbed of CO_2_ using Sodasorb^®^. The CO_2_-free air was then mixed with pure CO_2_ in precise quantities via digital mass flow controllers (Sierra Instruments, Monterey, CA, USA) and pumped into the sumps of experimental aquaria using venturi injectors. Two 170 L header tanks were also equilibrated with appropriate CO_2_ mixtures and were used to perform weekly water changes. Experimental tanks were sampled daily for total pH (pH_T_), temperature, salinity, $P_{{\mathrm{CO}}_{2}}$, and every 72 h for total alkalinity (T_A_), during the acclimation period (Table 1). For $P_{{\mathrm{CO}}_{2}}$ analysis, we followed the standard operating procedure as described in the Best Practices Guide (Riebesell *et al*., 2010) for the spectrophotometric determination of pH using m-Cresol Purple and measurement of total alkalinity via acid titration using a computer-controlled T50 Titrator (Mettler Toledo, Columbus, OH, USA). Temperature was measured with a calibrated digital thermocouple (Omega Engineering Inc., Stamford, CT, USA), and salinity was measured using a YSI 3100 conductivity meter (Yellow Springs, OH, USA). The program CO_2_ calc (Robbins *et al*., 2010), using the constants of Mehrbach *et al*. (1973) as refitted by Dickson and Millero (1987), was used to calculate all other carbonate parameters. Additionally, water quality assessments were made by testing for ammonia, nitrite and nitrate levels every 72 h. No detectable increase in nitrogenous waste was measured throughout each experiment.

**Table 1:** Mean ± SD values of temperature, salinity, pH (total scale), $P_{{\mathrm{CO}}_{2}}$ and total alkalinity over the course of the experiment

Treatment	Duration	Temperature (°C)	Salinity (ppt)	pH	*P*_CO_2__ (μatm)	Total alkalinity (μmol/kg solution)
Control	7 days	13.79 ± 0.74	33.46 ± 0.69	8.042 ± 0.034	406.15 ± 34.04	2286.51 ± 150.41
High	7 days	13.70 ± 0.80	33.73 ± 0.80	7.656 ± 0.055	1105.51 ± 152.72	2305.92 ± 41.29
Control	28 days	13.86 ± 0.32	33.48 ± 0.15	7.982 ± 0.045	475.05 ± 51.44	2273.10 ± 35.07
High	28 days	13.82 ± 0.31	33.53 ± 0.23	7.644 ± 0.034	1118.59 ± 93.20	2271.60 ± 22.85

### Evaluation of routine metabolic rate and critical oxygen tension

To establish RMR, oxygen consumption rates were measured using an intermittent respirometry system (Loligo systems, Denmark) described by Enzor *et al*. (2013). Fish were housed in 500 ml respirometry chambers fitted with internal acrylic sleeves (35–55 mm internal diameter) to restrict movement. These chambers were placed in covered 12-litre trays receiving a continuous flow of recirculating seawater (~115 litres total volume) held at treatment temperature (14°C) and control (~400 μatm) $P_{{\mathrm{CO}}_{2}}$. Fish were allowed to acclimate to the chambers for ~2–3 h with flush pumps running before metabolic rates were determined. Oxygen consumption was recorded over a 12 min period, followed by a 5 min flush period allowing reoxygenated seawater back into the chamber. Respiration rates (${\dot{M}}_{O_{2}}$) were measured according to Enzor *et al*. (2013). Oxygen consumption measurements were collected continuously over a 3 h period, with mean ${\dot{M}}_{O_{2}}$ values determined once no discernable chamber effect could be observed as indicated by no significant changes in oxygen consumption over a 1 h period and *r*^*2*^ values of >0.95 for the slope describing the rate of oxygen consumption for each measurement during this same time period.

Following the determination of RMR, we next evaluated how acclimation to elevated $P_{{\mathrm{CO}}_{2}}$ affected the hypoxia tolerance of *C. analis*. Critical oxygen tension was measured using the same intermittent respirometry system. Using a solenoid controlled by a galvanic O_2_ probe, pure nitrogen was gradually bubbled into the sump tank. Respiration rates were measured as described above as dissolved oxygen levels were lowered from 8 mg/l to severe hypoxia (<0.5 mg/l) over the course of ~3 h. Measurements of ${\dot{M}}_{O_{2}}$ continued on a 12 min record cycle followed by a 5 min flush cycle, until a discernable decrease in ${\dot{M}}_{O_{2}}$ from RMR was noticed. At this point, five additional flush cycles were conducted to ensure accurate calculations for $P_{O_{2}\mathrm{crit}}$. Upon completion of metabolic measurements, fish were humanely killed by immersion in MS-222 (Sigma Aldrich, St Louis, MO, USA). Gill tissues were excised, immediately frozen in liquid nitrogen and stored at −80°C until used for biochemical analysis.

### Spectrophotometric determination of Na^+^,K^+^-ATPase enzyme activity

Marine teleosts are known to down-regulate Na^+^,K^+^-ATPase (NKA) enzyme activity during hypoxic stress as a means to reduce oxygen-demanding processes. Therefore, to test whether differences in $P_{O_{2}\mathrm{crit}}$ were potentially related to NKA capacity in these fish acclimated to different $P_{{\mathrm{CO}}_{2}}$ levels, we quantified total NKA enzyme activity from gill tissue sampled from fish immediately after an acute hypoxic stress (the $P_{O_{2}\mathrm{crit}}$ analysis) using protocols that were optimized from McCormick (1993). Briefly, gill tissue (~50 mg) was homogenized in 200 μl of homogenization buffer (50 mM imidazole, 20 mM Na_2_EDTA, 300 mM sucrose, 0.2 mM phenylmethylsulfonyl fluoride and 5 mM beta mercaptoethanol, pH 7.0), followed by centrifugation of the homogenate at 375 g for 2 min. Supernatant was transferred to a new 1.5 ml microcentrifuge tube, where 2 μl of a 5 μg/μl alamethicin stock solution was added for improved membrane permeability, decreasing the risk of vesicle formation around enzymes. Samples were then incubated at the experimental temperature (14°C) in a water bath for 20 min. The ATPase activity was measured spectrophotometrically via the consumption of NADH in the presence or absence of the inhibitor ouabain. Uninhibited reactions were started by adding 15 μl of protein extract to 100 μl of MilliQ H_2_O and 2 ml assay cocktail [19 parts buffer (148 mM NaCl, 23.7 mM KCl, 7.74 mM MgCl_2_, 35.5 mM imidazole/Cl, 0.59 mM EGTA and 0.47 mM KCN, pH 7) and one part substrate solution (22.5 mM Na_2_ATP, 6.75 mM Na_2_NADH and 45 mM phosphoenolpyruvate)] in a 3 ml quartz cuvette. The cuvette was inverted to mix, and the rate of NADH disappearance was monitored at 340 nm. Inhibited reactions differed only in that 100 μl of 11.25 mM ouabain octahydrate was used instead of MilliQ H_2_O. Total Na^+^,K^+^-ATPase enzyme activity was calculated by subtracting the rate of NADH disappearance in the inhibited reaction from the activity rate of the uninhibited reaction.

### Statistics

We used a combination of analysis of variance (ANOVA) and covariance (ANCOVA) models to test for the effects of treatment and acclimation time on our responses. Given that mass has the potential to affect RMR (Clarke and Johnston, 1999) and thus $P_{O_{2}\mathrm{crit}}$ (Richards, 2011), variation in the size of individual fish was controlled for by using body mass as a covariate. Prior to analyses, we ensured that our data met normality and homoscedasticity assumptions. Specifically, we used an ANCOVA model to test for the effect of $P_{{\mathrm{CO}}_{2}}$ on routine metabolic rates at the 7 and 28 day acclimation times, using mass as covariate. In addition, we ran an ANCOVA model to test for the effect of acclimation time on routine metabolic rates within $P_{{\mathrm{CO}}_{2}}$ treatment groups, using mass as a covariate. Likewise, an ANCOVA model was also used in the evaluation of $P_{O_{2}\mathrm{crit}}$, with an effect of RMR or acclimation time and mass as a covariate. Two-way ANOVA was used in the evaluation of $P_{{\mathrm{CO}}_{2}}$ and acclimation time on Na^+^,K^+^-ATPase enzyme activity. Finally, given that the fish were collected at three different times over a 5 month period, we used an ANCOVA, with mass as a covariate, to test for the effect of collection date in order to rule out physiological differences related to seasonality. All statistical analyses were performed using the JMP (v11, SAS Cary, NC, USA) statistical analysis software.

## Results

### Seawater chemistry

Temperature, salinity and total alkalinity remained constant over the course of both experiments, with only pH and $P_{{\mathrm{CO}}_{2}}$ varying based on desired treatment levels (Table 1). Within both the 7 and 28 day acclimation groups, control and high-$P_{{\mathrm{CO}}_{2}}$ treatments differed significantly (Student's paired *t*-test; *P* < 0.05). Between acclimation times, only control $P_{{\mathrm{CO}}_{2}}$ and pH differed (*P* < 0.05). This difference, however, was <70 μatm $P_{{\mathrm{CO}}_{2}}$ or 0.06 pH units, well within natural variation of present-day ocean conditions.

### Routine metabolic rate

**Figure 1:**
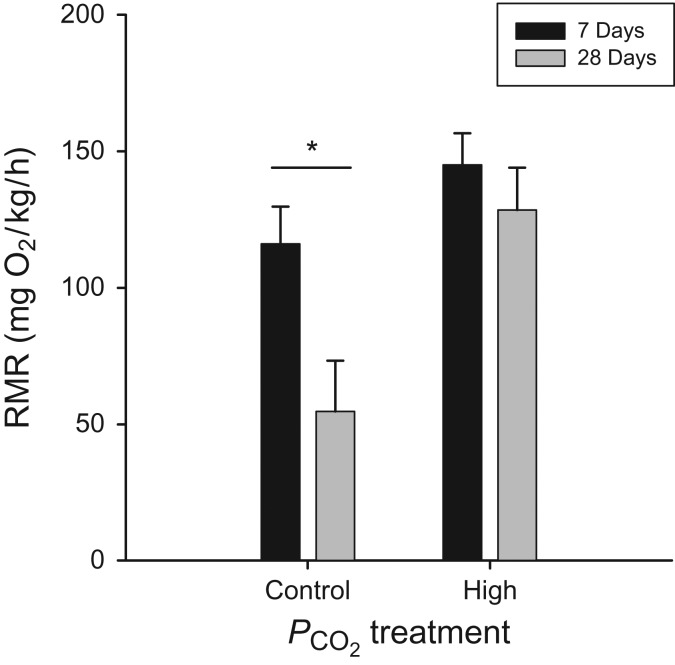
The routine metabolic rate (RMR) of *Clinocottus analis* when evaluating the effect of acclimation time in control and high-partial pressure of carbon dioxide ($P_{{\mathrm{CO}}_{2}}$) treatments, controlling for the mass of individual fish. Values are shown as least-squares means + SEM. *Statistical significance (*P* < 0.05) between time points within an acclimation group.

Within both the 7 and 28 day acclimation groups, *C. analis* acclimated to high $P_{{\mathrm{CO}}_{2}}$ showed significantly higher RMR (25.6 and 48.7%, respectively) compared with their control counterparts (ANCOVA; 7 days, *F*_(1,21)_ = 4.56, *P* = 0.047; 28 days, *F*_(1,11)_ = 20.002, *P* = 0.0009; Fig. 1). Interestingly, we found that RMR differed in the control treatments when compared between acclimation times (ANCOVA; control, *F*_(1,16)_ = 6.18, *P* = 0.024). Specifically, we observed a 52.9% decrease in RMR of 28-day-acclimated fish (Fig. 1). In contrast, RMR in the high-$P_{{\mathrm{CO}}_{2}}$ treatments remained the same at 7 and 28 days (ANCOVA; high, *F*_(1,16)_ = 0.67, *P* = 0.424). In addition, we also confirmed that mass affected RMR at both 7 and 28 days (ANCOVA; 7 days, *F*_(1,21)_ = 24.0045, *P* < 0.0001; 28 days, *F*_(1,11)_ = 16.0189, *P* = 0.0021). Lastly, we found no effect of collection date on RMR (ANCOVA; *F*_(2,34)_ = 2.358, *P* = 0.1099).

### Hypoxia tolerance

**Figure 2:**
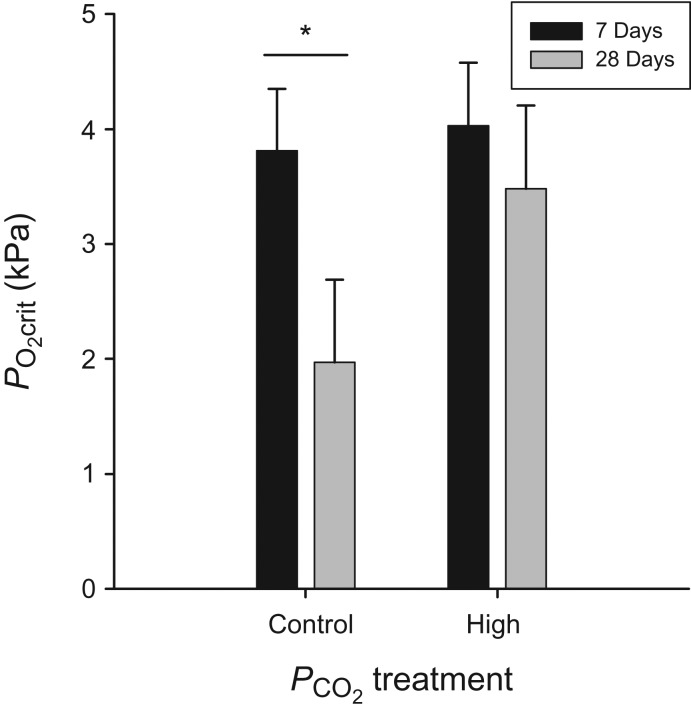
The hypoxia tolerance ($P_{O_{2}\mathrm{crit}}$) of *C. analis* when evaluating the effect of acclimation time in control and high-$P_{{\mathrm{CO}}_{2}}$ treatments, controlling for the mass of individual fish. Values are shown as least-squares means + SEM. *Statistical significance (*P* < 0.05) between time points within an acclimation group.

We observed no significant differences in $P_{O_{2}\mathrm{crit}}$ between treatments as an effect of RMR at 7 days; however, fish acclimated to the high-$P_{{\mathrm{CO}}_{2}}$ treatment displayed significantly higher $P_{O_{2}\mathrm{crit}}$ values after 28 days (ANCOVA; 28 days, *F*_(1,11)_ = 9.70, *P* = 0.0098). Although *C. analis* were no less hypoxia tolerant on day 7, by day 28 the fish acclimated to elevated CO_2_ appeared significantly less hypoxia tolerant than control fish as evidenced by a $P_{O_{2}\mathrm{crit}}$ that was 33.6% higher (Fig. 2). In addition, critical oxygen tension followed a pattern similar to that observed with RMR when fish within an treatment were compared across acclimation times. Within the control treatment, $P_{O_{2}\mathrm{crit}}$ decreased by 58% between fish acclimated for 7 and 28 days (ANCOVA; control, *F*_(1,16)_ = 6.91, *P* = 0.0182), although no discernable variation was observed within the high-$P_{{\mathrm{CO}}_{2}}$ treatment (ANCOVA; high, *F*_(1,16)_ = 0.32, *P* = 0.580; Fig. 2). Mass had no effect on $P_{O_{2}\mathrm{crit}}$ at 7 or 28 days (ANCOVA; 7 days, *F*_(1,21)_ = 1.52, *P* = 0.231; 28 days, *F*_(1,11)_ = 1.30, *P* = 0.278).

### Na^+^,K^+^-ATPase enzyme activity

**Figure 3:**
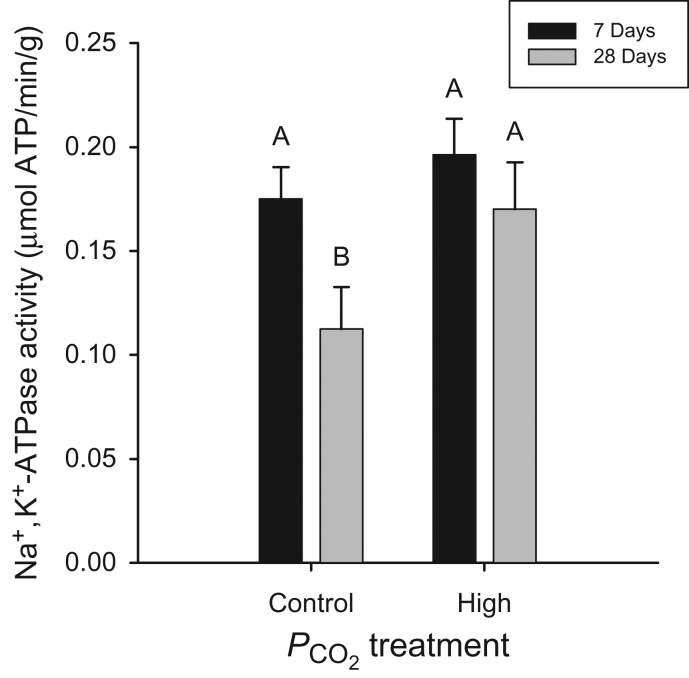
The total Na^+^,K^+^-ATPase activity when evaluating the effect of acclimation time in both control and high-$P_{{\mathrm{CO}}_{2}}$ treatments. Values are shown as adjusted means + SEM. Bars not indicated by the same letter are significantly different from each other (Tukey's HSD; *P* < 0.05).

Na^+^,K^+^-ATPase total enzymatic activity was quantified in gill tissue as a proxy for acid–base regulatory capacity in *C. analis*. We found a significant main effect of both $P_{{\mathrm{CO}}_{2}}$ level (two-way ANOVA; *F*_(1,28)_ = 4.731, *P* = 0.038) and acclimation time (two-way ANOVA; *F*_(1,28)_ = 7.0641, *P* = 0.012), Tukey's HSD multiple comparison testing showed that fish acclimated in the control treatment for 28 days displayed significantly lower NKA activity compared with all other treatments (Fig. 3). The variability in response between fish acclimated to the same experimental treatment for 7 and 28 days coincided with responses observed in RMR and $P_{O_{2}\mathrm{crit}}$. Within the control $P_{{\mathrm{CO}}_{2}}$ treatment, Na^+^,K^+^-ATPase activity decreased 35.6% by day 28; however, Na^+^,K^+^-ATPase activity remained constant in the high-$P_{{\mathrm{CO}}_{2}}$ treatment (Fig. 3).

## Discussion

Single-stressor studies for many marine animals, including fish, have generated a framework for understanding physiological and behavioural responses to the environment (Cooper *et al*., 2002; Kurihara, 2008; Munday *et al*., 2009a). However, the ecological relevance of results from these single-stressor studies may be limited, as it may be more common for organisms in the wild to experience shifts in multiple environmental parameters concomitantly, rather than a single parameter at a time. Although studies exploring the effects of multiple stressors on fish are less common, results of the few studies that have been conducted on other taxa suggest that the physiological response of a species to one environmental stressor can be contrary to the response to other stressors (Steckbauer *et al*., 2015). Such opposing responses raise the possibility that organisms might respond suboptimally during scenarios when multiple stressors change concomitantly (Gobler and Baumann, 2016).

In the present study, we set out to examine the vulnerability of *C. analis* to elevated $P_{{\mathrm{CO}}_{2}}$ levels (ocean acidification) in conjunction with hypoxia stress. Intertidal organisms that regularly experience steep gradients in $P_{{\mathrm{CO}}_{2}}$ are likely to have evolved greater capacities for offsetting the deleterious effects of short-term acidification (Seibel and Walsh, 2001, 2003). It is uncertain, however, whether those physiological adaptations will translate into a greater tolerance for chronic exposure to elevated $P_{{\mathrm{CO}}_{2}}$. Complexities in topography, biodiversity/density and local upwelling regimes within the rocky intertidal zone further confound this problem and indicate the need for experimental studies that use exposure regimes more complex than static $P_{{\mathrm{CO}}_{2}}$ manipulation. However, to date, no studies have investigated the amplified effects of multiple stressors on the behaviour or physiology of intertidal sculpins, even though research examining how these fishes respond physiologically to ocean acidification in an ecologically relevant context have the potential to provide crucial insights for predicting how acidification will impact rocky intertidal communities.

Our study examined how intertidal woolly sculpins were affected by exposure to differing levels of acidification coupled with environmental hypoxia. Both of these environmental variables are expected to be important stressors for intertidal sculpins experiencing a changing climate. Our results indicated there are increased metabolic costs associated with living in a more acidic ocean, which in turn could have consequences for hypoxia tolerance, as well as acid–base regulation. More specifically, the ability of sculpins to (i) avoid hypoxia through the down-regulation of oxygen-demanding cellular machinery directly conflicts with (ii) the defense against increasing carbon dioxide (acidity) stress via the up-regulation of oxygen-demanding Na^+^,K^+^-ATPase pumps. Perhaps most striking, however, is the fact that this study serves to highlight the degree of stress intertidal fishes are already surviving under, providing further support for the idea that these organisms are likely to be living near their physiological limits.

### Hypercapnia and metabolic rate

Previous studies have shown that physiological compensation to hypercapnia, through either acid–base regulation or ventilation, results in increased energetic debt for the organism (Perry and Gilmour, 2002, 2006; Evans *et al*., 2005). Heuer and Grosell (2014) point out that similar metabolic studies are highly limited and inconsistent in their results, but often operate under the assumption that fishes with capable acid–base regulatory mechanisms should increase basal metabolism when faced with hypercapnia. Indeed, the metabolic rates of marine animals are highly variable, with debates flexing around the importance of anatomical features, ecological function and environmental constraints (Seibel and Drazen, 2007). For *C. analis*, RMR was significantly higher in fish acclimated to elevated $P_{{\mathrm{CO}}_{2}}$ in both the short (7 day) and longer-term (28 day) experiments, with the highest measured RMR coming after 28 days of acclimation to a $P_{{\mathrm{CO}}_{2}}$ of ~1100 μatm. Metabolic responses consistent with our results have also been found in other fish species, including species from tropical (Munday *et al*., 2009b) and polar ecosystems (Enzor *et al*., 2013), where either total aerobic scope was decreased or RMR increased. Our results suggest that the intertidal environmental constraints most likely to be associated with fluctuating abiotic conditions, including hypercapnia, at least in part could significantly drive metabolic responses within *C. analis*.

Although the RMR of *C. analis* acclimated to high $P_{{\mathrm{CO}}_{2}}$ was significantly higher than that of fish acclimated to the control treatment (~400 μatm), we observed no differences across time points within the high-$P_{{\mathrm{CO}}_{2}}$ acclimation groups. This differs from the metabolic response observed in Antarctic notothenioids, which initially display a sharp increase in RMR after 7 days of acclimation to elevated $P_{{\mathrm{CO}}_{2}}$, yet quickly return to basal ${\dot{M}}_{O_{2}}$ levels within 28 days of acclimation (Enzor *et al*., 2013). Unexpectedly, ${\dot{M}}_{O_{2}}$ values for *C. analis* acclimated to ~400 μatm $P_{{\mathrm{CO}}_{2}}$ showed a significant decline (~52.9%) after 28 days in experimental conditions compared with the RMR of fish acclimated to the control treatment for only 7 days. We postulate that placing these animals in a static experimental environment alleviated the high levels of stress routinely encountered in the intertidal zone owing to the absence of tidal or daily cycles, thus reducing the metabolic costs typically associated with life in a tide pool. Despite being periodically replenished with ambient seawater, tide pools in the marine intertidal zone are known to display extreme swings in abiotic parameters, such as dissolved oxygen concentration, pH, salinity and temperature (Truchot and Duhamel-Jouve, 1980; Morris and Taylor, 1983; Bridges *et al*., 1984). It is plausible that the likelihood of experiencing these extreme environmental shifts is high enough to force these fish to maintain cellular defense mechanisms at a relatively high constitutive level. Osmoregulation and the heat shock response are crucial cellular defense mechanisms routinely employed by organisms that exist in highly variable environments (Kültz, 2005; Fangue *et al*., 2006; Evans and Somero, 2008; Somero, 2010; Tomanek, 2010), both of which can come at significant energetic cost to the organism (Goolish and Burton, 1989; Iwama *et al*., 1998; McAllen and Taylor, 2001; Tomanek and Zuzow, 2010). Relaxation of these and other costly cellular defense mechanisms could explain the observed reduction in RMR when significant environmental variation is removed. For example, field studies involving *Mytilus californianus* have shown key indications of both aerobic and anaerobic capacity, which are elevated in populations routinely exposed to variation in pH and dissolved oxygen as a result of periodic upwelling. These same metabolic indicators are routinely lower in populations that do not experience significant variations in these abiotic parameters (Dahlhoff and Menge, 1996). Furthermore, wave-exposed regions within the rocky intertidal zone that provide relief from thermal stress are characterized by mussels with lower indicators of cellular stress (heat shock proteins) and increased RNA-to-DNA ratios (a proxy for growth potential; Dahlhoff and Menge, 1996; Dahlhoff *et al*., 2001, 2002).

By removing these natural environmental stressors in our control treatments, we would expect to see indications of the relaxation of energetic constraints, particularly when examining the downstream effects of RMR on hypoxia tolerance, the role of NKA activity in acid–base regulatory capacity and the behavioural responses to hypoxia. For instance, some fish species like *C. analis* can meet metabolic oxygen needs during bouts of hypoxia through behavioural responses, such as aerial surface respiration (Congleton, 1980; Watters and Cech, 2003). However, we were unable to observe these behavioural responses in *C. analis* individuals that had been acclimated to either the control or high-$P_{{\mathrm{CO}}_{2}}$ treatments, even when exposed to severe anoxia for extended periods (data not shown). The absence of these behaviours suggests that these treatments never entailed a metabolic cost large enough to elicit the need to perform aerial surface respiration even in a low-oxygen environment.

### Hypoxia tolerance and acid–base regulation

Similar to our observations with respect to metabolic rate, *C. analis* did not exhibit any differences in hypoxia tolerance ($P_{O_{2}\mathrm{crit}}$) or NKA activity between treatments after 7 days of acclimation to elevated $P_{{\mathrm{CO}}_{2}}$. Although similar acid–base regulatory studies are absent among intertidal fish, others have observed increases in NKA for marine teleosts when challenged with hypercapnia (Deigweiher, 2009; Deigweiher *et al*., 2010). Thus, our findings confirm the aforementioned idea that intertidal animals, particularly vertebrate fish, are likely to have the capacity to offset short-term bouts of acidification or hypercapnia. This comes as no surprise because seasonal upwelling, which acts as a selective pressure along the California coast, often brings these animals in contact with corrosive waters (Feely *et al*., 2008). Our data, however, do suggest that longer-term acclimation to high $P_{{\mathrm{CO}}_{2}}$ may incur a significant physiological cost to *C. analis*, because we found a significant difference in hypoxia tolerance and acid–base regulatory capacity between control and high-$P_{{\mathrm{CO}}_{2}}$ treatment groups after 28 days of acclimation. Alternatively, the high-$P_{{\mathrm{CO}}_{2}}$-treated fish could potentially be experiencing acute alkalosis during the RMR/$P_{O_{2}\mathrm{crit}}$ measurements; thus, the lack of change observed in this group between the 7 and 28 day time points may be reflective of efforts to compensate for changes in internal CO_2_ levels by changing HCO_3_^−^ and blood pH. However, previous studies looking at the effects of acute alkalosis on the hypoxia response in trout have shown that initial blood alkalosis lowered the partial pressure of oxygen threshold at which the hypoxia response begins, effectively increasing their hypoxia tolerance (Thomas *et al*., 1992). As we did not observed a similar decrease in $P_{O_{2}\mathrm{crit}}$ for fish acclimated to the high-$P_{{\mathrm{CO}}_{2}}$ treatment on the hypoxia tolerance, the effect we observed here is not likely to be related to acute alkalosis.

As seen with measurements of RMR, we again saw no difference in total acid–base regulatory capacity or hypoxia tolerance in fish acclimated to elevated $P_{{\mathrm{CO}}_{2}}$ across time points. However, *C. analis* acclimated to control treatments showed significantly lower NKA levels and were more hypoxia tolerant in 28 day acclimated groups compared with 7 day acclimated groups. These data further support the idea that fish acclimated to the high-$P_{{\mathrm{CO}}_{2}}$ treatment display physiological capacities that reflect the basal requirements of life in the intertidal zone. More specifically, our data confirm that acclimation to future ocean acidification scenarios sets increased demands on acid–base balance in these fish via the NKA pump and that this, in turn, comes at a metabolic cost that is equivalent to the maintenance costs associated with the current natural variation that occurs in rocky tide pools. For *C. analis*, it is unclear whether the lack of change in NKA capacity over 28 days suggests that these fish have reached their maximal capacity to respond. However, even if these fish maintain sufficient capacity to elevate NKA activity further, it is possible that they would see further reductions in hypoxia tolerance.

Our data suggest that increases in $P_{{\mathrm{CO}}_{2}}$ are compensated, in part, through changes in acid–base regulatory capacity, and this may indirectly impact hypoxia tolerance in these fish. Routine metabolic rate has previously been shown to have a direct impact on $P_{O_{2}\mathrm{crit}}$ (Richards, 2011). As such, the increased metabolic rate necessary to maintain NKA capacity in high-$P_{{\mathrm{CO}}_{2}}$ conditions has resulted in a reduction in hypoxia tolerance. This reduced tolerance is increasingly problematic when considering the exacerbating effect of ocean acidification on natural fluctuations in tide pool carbon dioxide levels. For example, Truchot (1988) demonstrated that at nighttime, when respiration rates are high, $P_{{\mathrm{CO}}_{2}}$ levels in tide pools can exceed 3000 μatm. As global $P_{{\mathrm{CO}}_{2}}$ averages rise, it is likely that tide pools with little photosynthetic activity could reach similar values during daytime hours, creating increased oxygen demands on these systems. Field observations (data not shown) confirm that many *C. analis* reside in high intertidal pools where algal cover is limited, thus they may find themselves continuously challenged to meet the metabolic demands of life in a highly variable environment.

### Conclusions

As we evaluate the ability of marine species to respond to environmental stress associated with climate change, it becomes increasingly important to consider the effects of multiple naturally occurring concomitant stressors. Our study highlights two naturally occurring stressors, hypoxia and ocean acidification, that may require trade-offs between hypoxia tolerance and acid–base regulation capacity. It is likely that climate change will further exacerbate these natural stressors, emphasizing the need for scientific studies to address these challenges. To our knowledge, no studies have looked at the effects of these interacting climate-change-related stressors on intertidal fish; therefore, the present study provides crucial insight into an important component of rocky intertidal ecosystems.

The limited data available from multistressor studies on marine fishes (e.g. Munday *et al*., 2009b; Enzor *et al*., 2013; Ferrari *et al*., 2015) point to a crucial need to consider the broader effects that climate change may have on the overall condition of these animals. It has become evident that even though an organism may possess the physiological tools and capacity to withstand predicted changes in ocean chemistry, that physiological plasticity is likely to come at a significant cost that will have impacts on behaviour and/or energy allocation, which may alter ecosystem dynamics. Lastly, these studies have helped to highlight that our current understanding of the susceptibility of teleost fish to changes in ocean pH may be underestimated. Consequently, future conservation strategies must consider multistress experiments when assessing the impacts of global climate change on species distributions and population dynamics.
